# Recovery of anosmia in hamsters infected with SARS-CoV-2 is correlated with repair of the olfactory epithelium

**DOI:** 10.1038/s41598-021-04622-9

**Published:** 2022-01-12

**Authors:** Rachel A. Reyna, Megumi Kishimoto-Urata, Shinji Urata, Tomoko Makishima, Slobodan Paessler, Junki Maruyama

**Affiliations:** 1 Department of Pathology, University of Texas Medical Branch, Galveston, TX 77555 USA; 2 Department of Otolaryngology, University of Texas Medical Branch, Galveston, TX 77555 USA

**Keywords:** SARS-CoV-2, Infection

## Abstract

Severe acute respiratory syndrome coronavirus-2 (SARS-CoV-2) is responsible for a pandemic affecting billions of people worldwide. Apart from the extreme global economic impact, the pandemic will likely have a lasting impact through long-term sequelae not yet fully understood. Fully understanding the mechanisms driving the various symptoms and sequelae of SARS-CoV-2 infection will allow for the eventual development of therapeutics to prevent or treat such life-altering symptoms. In this study, we developed a behavioral test of anosmia in SARS-CoV-2-infected hamsters. We find a moderately strong correlation between the level of anosmia and the score of histological damage within the olfactory epithelium. We also find a moderately strong correlation between the level of anosmia and the thickness of the olfactory epithelium, previously demonstrated to be severely damaged upon infection. Thus, this food-searching behavioral test can act as a simple and effective screening method in a hamster model for various therapeutics for SARS-CoV-2-related anosmia.

## Introduction

In December of 2019, the first cases of what would quickly become a global pandemic were reported out of Wuhan, China. Since then, the novel severe acute respiratory syndrome coronavirus 2 (SARS-CoV-2) quickly spread around the world, causing millions of cases of the coronavirus disease 2019 (COVID-19). At the time of writing, the United States alone has reported over 47 million cases^1^. Globally, SARS-CoV-2 is responsible for over 255 million cases and over 5.1 million deaths^1^. In many places, total shutdowns were ordered with the goal of preventing the spread of the virus. However, countries around the world are still struggling to balance concerns of public health with potentially crippling economic repercussions. Understanding potential long-term effects of infection is crucial for successful global recovery.

While the majority of SARS-CoV-2 infections are mostly asymptomatic or present with mild flu-like symptoms, in severe cases, patients may develop severe pneumonia, requiring urgent medical intervention^2^. One of the most well-documented symptoms of COVID-19 is anosmia, as it is prevalent in all degrees of infection, mild to severe^3–5^. Of note, SARS-CoV-2 can cause anosmia at a high rate as compared to other infectious organisms^6^. Previous studies have indicated that this olfactory dysfunction occurs in 33.9–85.6% of all COVID-19 cases^7–10^. Although some studies indicate that the anosmia may subside after 3–4 weeks^11,12^, the long-term consequences of this destruction of olfactory epithelium remains unknown.

Syrian golden hamsters are an excellent small animal model to study SARS-CoV-2 infection due to their low mortality, marked pathology, and competent immune response^13–15^. We have previously reported that SARS-CoV-2-infected hamsters demonstrate severe damage to the olfactory epithelium (OE) of nasal turbinates as early as 3 days post-infection (dpi)^16^. We also reported that the OE was indeed regenerating, with significant recovery of thickness by 21 dpi^16^. Although this finding is consistent with clinical reports of COVID-19 patients eventually recovering their sense of smell, no study has directly correlated this OE regeneration with a recovery of olfactory function or definitively outlined a mechanism of OE destruction leading to anosmia. With an increased understanding of this mechanism, therapeutics can be developed that will prevent or treat anosmia in COVID-19 patients. While screening therapeutic or preventative effectiveness against SARS-CoV-2 infection is possible by histopathological analysis on OE in infected hamsters, this technique is often extremely time consuming and difficult. In this study, we developed a simple behavioral test with SARS-CoV-2-infected hamsters to assess olfactory function directly. This simple behavioral test, with the results correlated to OE damage, has significant translational potential; therapeutic or preventative efficacy can be determined much more quickly.

## Results

### SARS-CoV-2 challenge and olfactory behavioral test in hamsters

**Figure 1:**
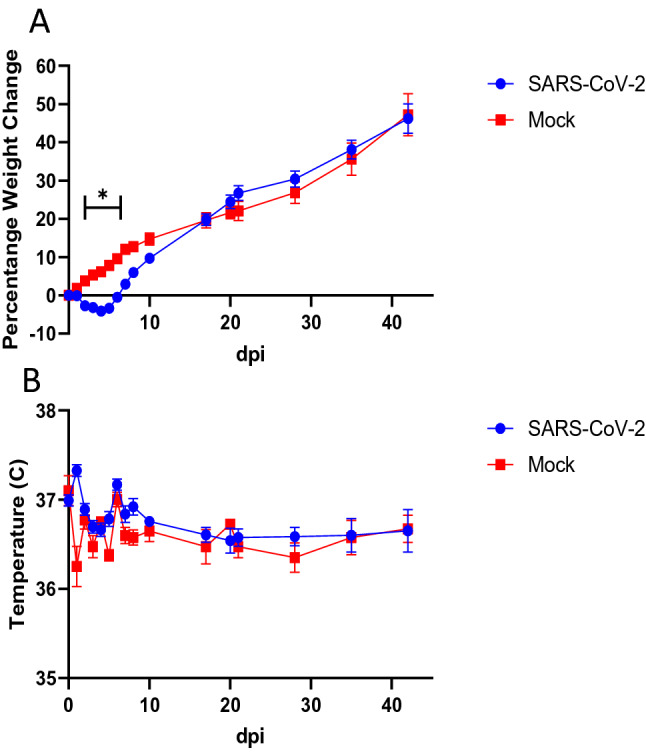
Body weight and temperature change in hamsters. Weight change (**A**) and temperatures (**B**) for SARS-CoV-2-inoculated (n = 32) versus mock-inoculated (n = 4) Syrian golden hamsters were shown in average ± SEM/ SARS-CoV-2-inoculated hamsters were euthanized for sample collection at 2, 3, 5, 8, 17, 21, 35, and 42 dpi (n = 4 each). Significant differences between SARS-CoV-2- and mock-inoculated groups at each timepoint were determined by a two-way ANOVA followed by Fisher’s LSD test. *p < 0.05.

**Figure 2:**
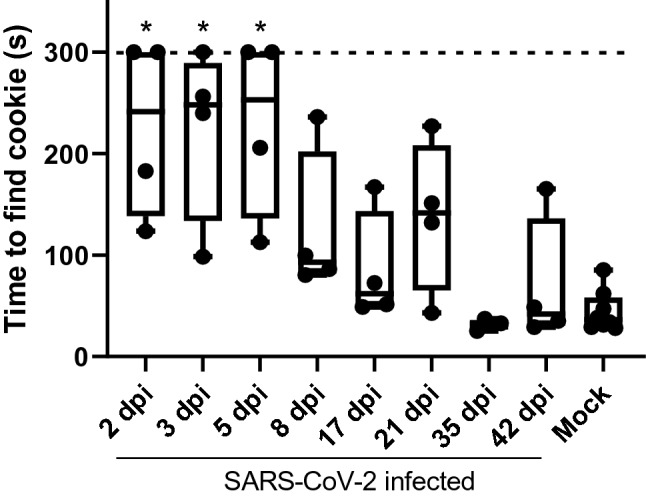
Time (s) taken for hamsters to find the hidden cookie. The time required for hamsters to find the hidden cookie were shown in box plots. The broken line indicates the cut off value of measurement (300 s). Significant differences compared to mock group were determined by one-way ANOVA followed by Dunnett’s post hoc test. *p < 0.05. Mock *n* = 8; all infected groups *n* = 4.

All hamsters inoculated intranasally with 10^5^ 50% tissue culture infectious dose (TCID_50_) of SARS-CoV-2 survived infection, featuring only a mild, transient weight loss and no development of fever (Fig. 1) as previously described. SARS-CoV-2-inoculated hamsters were tested for anosmia at 2, 3, 5, 8, 17, 21, 35, and 42 dpi. Mock-inoculated hamsters were tested for a baseline at 2 dpi and again at 42 dpi. The behavioral test consisted of burying a Teddy Graham cookie in one corner of a cage and measuring the time required for a hamster to find the cookie. Hamsters tested at 2, 3, and 5 dpi required significantly increased time to find the hidden cookie in comparison to the mock group (Fig. 2). Of note, some infected hamsters did not find the cookie within the maximum time provided in this study (300 s), and they did not show interest in the cookies, even when placed directly in front of them. Although not significantly different, the times to find the cookie of the remaining groups gradually trended shorter towards that of mock group, indicating a slow recovery of the sense of smell.

### Histopathological analysis of olfactory epithelium

**Figure 3:**
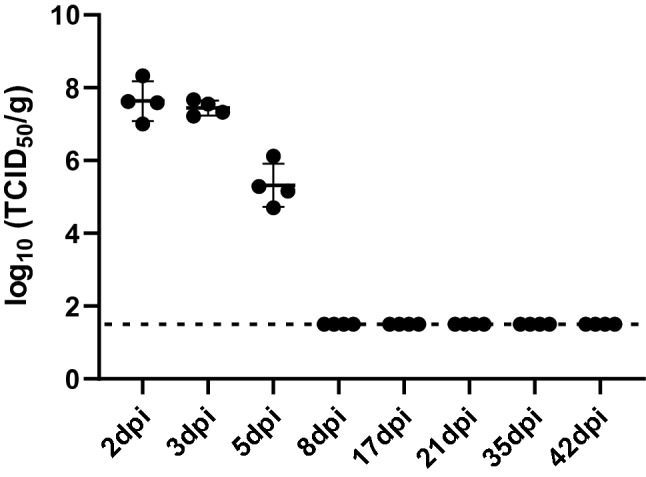
Virus titers in the lungs of SARS-CoV-2-inoculated hamsters. Averages and SEMs of virus titers in the lungs were plotted. The broken line indicates the limit of detection (< 1.50 log_10_ TCID50/g). All groups *n* = 4.

**Figure 4:**
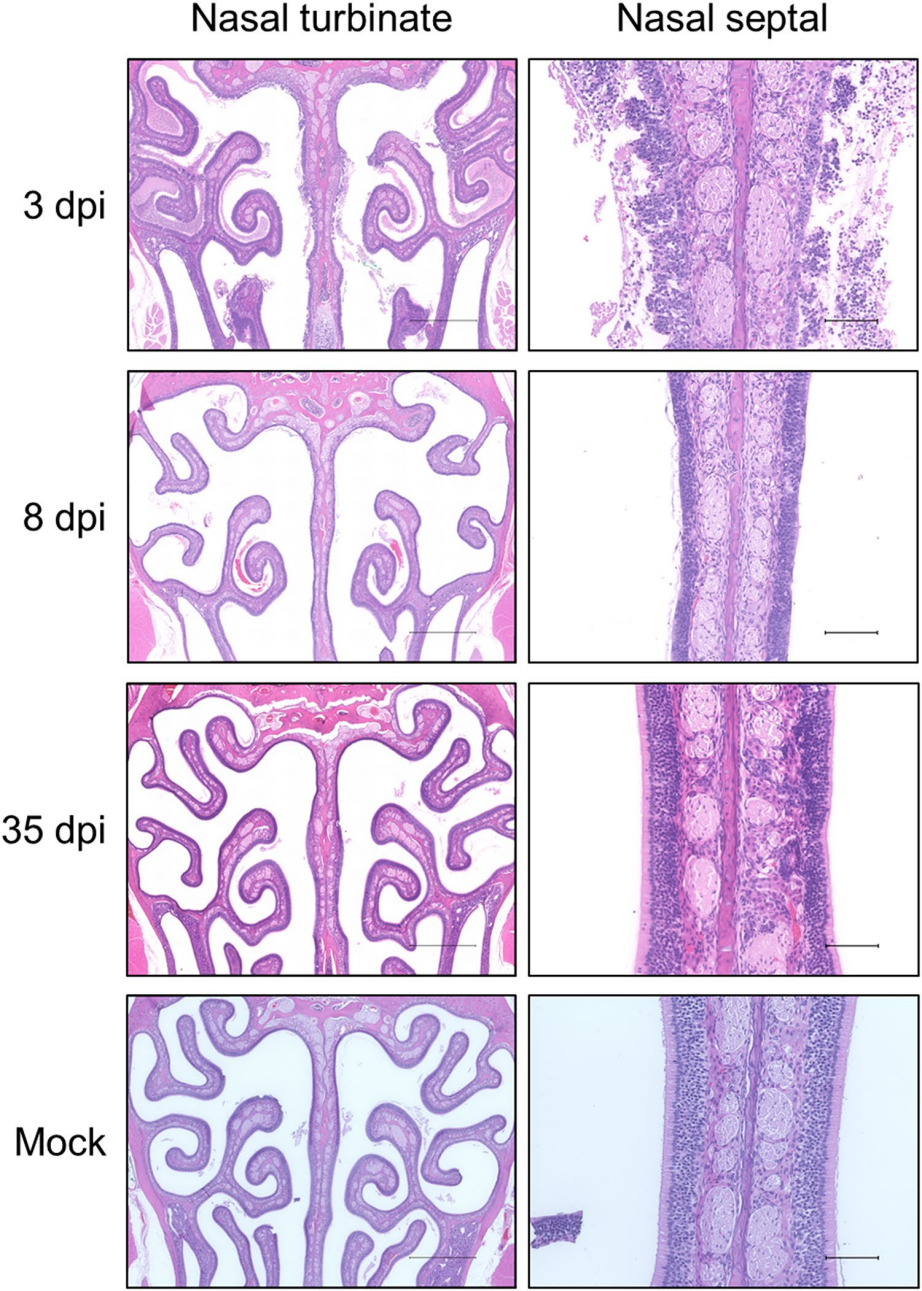
Representative histology of nasal turbinate in hamsters. Significant olfactory epithelial damage and nasal discharge is evident through 8 dpi in samples. Scale bars: 1 mm (left), 100 µm (Right).

**Figure 5:**
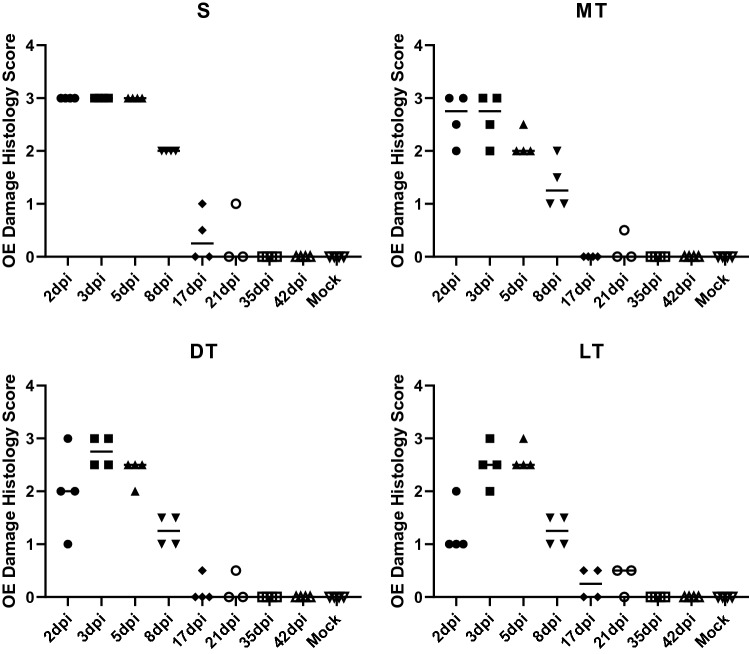
Histology scores of nasal turbinate. Scoring of gross histopathological changes of the olfactory epithelium in the four nasal turbinate regions: S—nasal septal, MT—medial turbinate, DT—dorsal turbinate, LT—lateral turbinate. 0—no damage (normal), 1—mild damage (damage reaches only through epithelial layer; basal layer remains untouched), 2—moderate damage (up to 75% of damage reaches basal cells), 3—severe damage (over 75% of damage reaches basal cells). Scores were averaged between two independent reviewers. 21dpi *n* = 3; all other groups *n* = 4.

**Figure 6:**
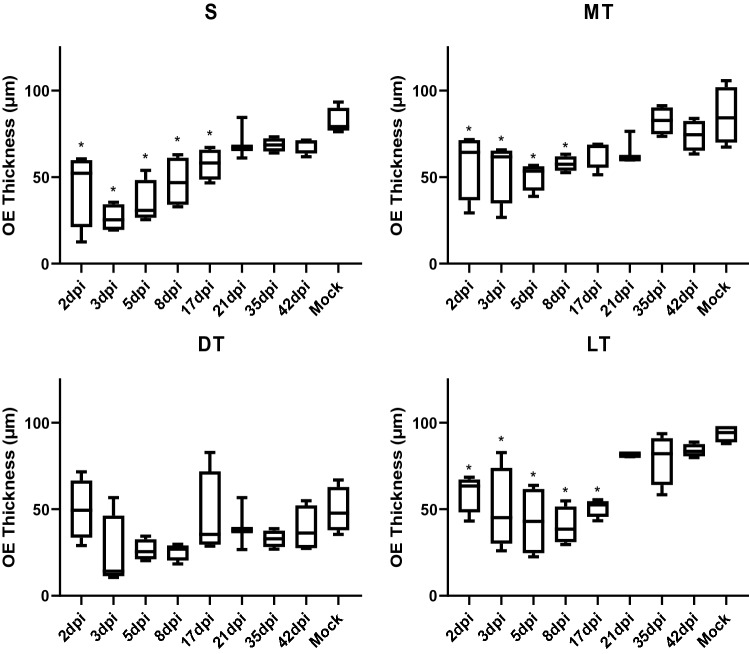
Olfactory epithelium thickness in nasal turbinate. Box plots of the thickness (μm) of the olfactory epithelium (OE) in the four nasal turbinate regions were shown: S—nasal septal, MT—medial turbinate, DT—dorsal turbinate, LT—lateral turbinate. All results the average of two independent measurements. 21 dpi *n* = 3; all other groups *n* = 4. * = *p* < 0.05.

After behavioral testing, lung and nasal samples were collected for viral titration and histology, respectively. As expected, virus was detected from lungs with a high titer at 2 and 3 dpi, and decreased at 5dpi, and then virus was cleared from the lungs by 8 dpi (Fig. 3). Histology was performed on coronal nasal samples (Fig. 4) in order to quantify damage to the OE using two separate approaches: scoring of gross histopathological damage (Fig. 5) and direct measurement of OE thickness (Fig. 6). A marked increase in histological scores were seen in all four turbinate regions through 8dpi (Fig. 5). OE thickness was significantly reduced through 17 dpi for the nasal septum (S) and lateral turbinate (LT), and 8 dpi for the medial turbinate (MT) (Fig. 6). OE thickness was not significantly impacted in the dorsal turbinate (DT) (Fig. 6).

To determine whether correlations exist between the histopathological change in the OE and the time to find the hidden cookie, we checked Pearson’s correlation between the time to find the cookie, the OE thickness, and the histology score in the OE (Table 1). We see a significant, moderately strong positive correlation between the time to find the cookie and the histology score. We also see a significant, mildly negative correlation between the time to find the cookie and the OE thickness.

**Table 1:** Pearson correlation values of histology, OE measurements, and time to find the cookie.

	Thickness				Histopathology score			
	S	MT	DT	LT	S	MT	DT	LT
Pearson's *r*	− 0.57561	− 0.35408	− 0.3295	− 0.47092	0.743831	0.7385	0.762914	0.722599
*p* value	0.000126	0.027	0.040524	0.00248	5.76E-08	8.02E−08	1.64E−08	2.06E−07

A Pearson correlation was performed for the time taken to find the hidden Teddy Graham cookie (s), the olfactory epithelium (OE) thickness (μm) of the four individual nasal turbinate regions: S—nasal septal, MT—medial turbinate, DT—dorsal turbinate, LT—lateral turbinate), and the histology scoring of each turbinate.

## Discussion

SARS-CoV-2-associated anosmia is perhaps one of the most well-known symptoms of COVID-19. While our previous work indicates that the anosmia is likely temporary, due to the gradual recovery of the severely damaged OE^16^, the long-term consequences of this anosmia remains unknown. Additionally, whether therapeutics can be developed to treat those suffering anosmia remains to be seen. Syrian golden hamsters are an excellent animal model through which to screen vaccines and therapeutics for SARS-CoV-2 infection^13^. Not only does infection consistently produce a transient acute phase of disease featured by a mild weight loss, but the hamsters themselves are outbred and immunocompetent, making the model much more relevant to COVID-19 in humans. Previous histology studies focusing on the OE damage within these hamsters requires intensive and time-consuming techniques. Moreover, as human histological samples are difficult to obtain, a behavioral test that reproduces the large amounts of human anosmia data is desirable. Thus, the objective of this study was to further develop a simple behavioral test to assess olfactory function directly to aid in the screening process of anosmia therapeutics.

As expected, our SARS-CoV-2-inoculated hamsters developed a transient weight loss with viral titers cleared from the lungs by 8 dpi (Figs. 1 and 3). This is consistent with previous studies, but one additional explanation for why the hamsters lose weight may be that they are simply unable to smell their food due to the significant OE damage (Fig. 6). This weight loss paired with the increase in time to find the hidden cookie (Fig. 2), the marked pathology in the nose, and the significant thinning of the OE emphasize the effectiveness of the Syrian golden hamster as an anosmia model for COVID-19.

One of the main objectives of this project was to assess the olfactory function directly by using a behavioral test for the confirmation of anosmia in a hamster COVID-19 model. Ideally, the time taken for each hamster to find the hidden cookie would correlate with OE damage; longer searching times would correlate with OE damage. We performed a more intensive correlation test, using Pearson’s correlation test (Table 1). We found a significant and moderately strong positive correlation between the time taken to find the cookie and the histology score for each turbinate region. This is to be expected, as the visible histological damage requires at least 8 dpi to be cleared, by which the behavioral results are no longer significantly different. Additionally, we see a significant and moderate negative correlation between the time to find the cookie and the OE thickness of each turbinate region. Again, this result was expected as regeneration of the OE thickness should be related to the recovery of the sense of smell. Overall, based on our histology scores and thickness in each OE region, the nasal septal region (S) is the most consistently correlated with the olfactory behavior tests. This indicates S may be the most suitable region for future histopathological analyses to asses OE damages induced by SARS-CoV-2 infection. It is important to note that the receptor for SARS-CoV-2, angiotensin-converting enzyme 2 (ACE2), may not be equally expressed between the nasal turbinate regions, accounting for the differences in severity of damage seen upon histology. Previous work indicates that the septal and dorsal regions may express higher levels of ACE2, rendering these zones more susceptible to SARS-CoV-2 infection^17^. Additionally, nasal turbinate scrolls are possibly more physically difficult for the virus to reach. However, as the nasal septal region is physically supported well by other supporting tissues and thicker bone structures, it is more resistant to acquired damage during sample preparation, as compared to the other regions. Thus, we propose that the nasal septal region will be a good target for future histopathological analysis of OE damage in COVID-19 patients.

While we find that histological damage moderately correlates with olfactory function, previous work using a chemically induced anosmia has indicated that over 90% OE destruction is required before anosmia occurs^18^. This strengthens our claim that a behavioral test is more desirable to detect functional changes during screening of vaccines and therapeutics than relying solely on histology and provides further insight into a potential mechanism.

Previous research indicates that SARS-CoV-2-related anosmia may be due to infection of non-neuronal cells within the OE; most olfactory sensory neurons (OSNs) do not express ACE2^17,19^. These studies indicate that anosmia may be linked to infection of the sustentacular cells supporting the olfactory system, resulting in a rapid and severe deciliation of the OE^19,20^. Without cilia to detect odorants, the resulting anosmia occurs^20^. According to Butowt and von Bartheld, this is the most likely mechanism for this anosmia^21^. Based on our results, this deciliation mechanism is also likely. Comparing our results to that of others, there is a similar timeline of damage and regeneration. Significant anosmia occurs between 2 dpi through 5 dpi, followed by a gradual trend of recovery, with hamsters regaining seemingly normal olfactory function by 35 dpi (Fig. 2). Similarly, previous work in a Syrian golden hamster model demonstrates peak deciliation occurring as early as 2 dpi with OE recovery and functional cilia appearing back around 14 dpi^20^. Further work should be conducted directly examining any correlations between the ciliation of the OE and olfactory function, as measured by using this food behavioral test.

In the case of hamsters that did not find the cookie within the maximum time frame (300 s), we confirmed that hamsters were unable to detect the cookie by either directly handing it to them or by placing it immediately in front of them and watching for their reaction. All hamsters that had significantly higher search times were unable to find, or were completely uninterested in, the cookies in both of these situations. This fact helps to emphasize that these hamsters indeed developed severe anosmia. Thus, this study indicates that this anosmia behavioral test is suitable for direct assessment of the olfactory function. Moreover, this behavioral test can be used as a fast and simple indicator of the efficiency of vaccines or therapeutics against SARS-CoV-2 infection in the hamster model. This test will contribute to the speedy development of therapeutics to help aid those suffering from anosmia related to COVID-19.

## Methods

### Cells and virus

Vero E6 cells were maintained using Dulbecco’s modified Eagle’s medium (DMEM) supplemented with 10% fetal bovine serum (FBS), 1% penicillin–streptomycin, and L-glutamine. SARS-CoV-2 (USA/WA-1/2020) was received from the World Reference Center for Emerging Viruses and Arboviruses (WRCEVA) at UTMB and propagated in Vero E6 cells with DMEM supplemented with 2% FBS. Cell culture supernatant was stored in the − 80 °C freezer until use.

### Animal experiments

Five- to six-week-old female Syrian golden hamsters were purchased from Charles River and inoculated intranasally with 100 μL of either 10^5^ TCID50 of SARS-CoV-2 diluted in phosphate-buffered saline (PBS) or PBS as a mock control. Body temperatures were measured using subcutaneously implanted BMDS IPTT-300 transponders and the DAS-8007 transponder reader from Bio Medic Data Systems. Body weights were measured with an Ohaus CX1201 Portable Scale. All hamsters were housed in the animal biosafety level-2 (ABSL-2) and ABSL-3 facilities within the Galveston National Laboratory at the University of Texas Medical Branch (UTMB). A high-flow rate of CO_2_ followed by thoracotomy were used at the time of euthanasia. All animal studies are reviewed and approved by the Institutional Animal Care and Use Committee at UTMB and are conducted according to the National Institutes of Health guidelines. This study is reported in accordance to ARRIVE guidelines (https://arriveguidelines.org/arrive-guidelines).

### Behavioral testing

Our behavioral test was developed using the buried food test based on Yang et al. in 2009^22^. On each sampling day (2, 3, 5, 8, 17, 21, 35, and 42 dpi), four hamsters were tested for any signs of anosmia. Testing occurred in the afternoon at approximately the same time each testing day. An empty housing cage was prepared with at least 3 cm of bedding. In one corner, a honey-flavored Teddy Graham (Nabisco) was buried about 1 cm below the surface of the bedding. In the corner diagonal to the buried cookie, a hamster was placed, the lid closed, and a timer started. The timer was stopped once the hamster had revealed and grasped the cookie, and the time recorded. All hamsters were given a maximum of 5 min (300 s) to find the cookie.

### Viral titration

Collected lung samples were homogenized with DMEM supplemented with 2% FBS to make a 10% homogenate. These samples were then diluted tenfold serially and inoculated into Vero E6 cells on 96-well plates. Infected cells were allowed to incubate for 72 h at 37 °C with 5% CO_2_, then fixed with 10% formalin and stained with 0.25% crystal violet to visualize the presence of cytopathic effect (CPE). TCID_50_ values were then calculated and recorded using the Reed and Muench method^23^.

### Histological analysis

At the time of sampling, collected muzzles were fixed in 10% buffered formalin for 7 days before removal from the BSL-3 facilities. Samples were prepared for histological analysis as previously described^16^. Briefly, olfactory bulbs and nasal tissue including the olfactory epithelium (OE) were extracted from the skull and decalcified with EDTA (10% w/v) and embedded in paraffin. Thin sections with 5 μm thickness were mounted on glass slides and stained with Hematoxylin and Eosin. To analyze the OE, coronal sections of the OE were divided into four areas along zonal organization as previously described^16^ and two independent analyses were performed examining the histopathology of the OE for each group. Each of the four turbinate zones were examined and assigned a pathology score: 0—no damage (normal), 1—mild damage (damage reaches only through epithelial layer; basal layer remains untouched), 2—moderate damage (up to 75% of damage reaches basal cells), or 3—severe damage (over 75% of damage reaches basal cells). Scores were averaged between the two reviewers. To investigate the changes in OE thickness, two independent measurements were performed for the four different nasal turbinate regions as previously described^16^.

### Statistical analysis

Statistical analyses were performed using the GraphPad Prism software (ver 9.1.2). Statistical significance for weight changes between SARS-CoV-2-infected and uninfected hamsters were determined using a two-way ANOVA followed by Fisher’s LSD test. Statistical significance for the behavioral test and olfactory epithelium thickness was determined using a one-way ANOVA followed by Dunnett’s post hoc test. Correlations were determined using a Pearson correlation test. *p* < 0.05 was deemed statistically significant.
